# Water content for clot composition prediction in acute ischemic stroke

**DOI:** 10.1371/journal.pone.0304520

**Published:** 2024-05-24

**Authors:** Kenichi Sakuta, Taichiro Imahori, Amir Molaie, Mahsa Ghovvati, Neal Rao, Satoshi Tateshima, Naoki Kaneko

**Affiliations:** 1 Department of Radiological Sciences, David Geffen School of Medicine at UCLA, Los Angeles, CA, United States of America; 2 Department of Neurology, Jikei University School of Medicine, Tokyo, Japan; 3 Department of Neurology, David Geffen School of Medicine at UCLA, Los Angeles, CA, United States of America; 4 Department of Neurosurgery, Kitaharima Medical Center, Hyogo, Japan; Global Health Neurology Lab / NSW Brain Clot Bank, NSW Health Pathology / Liverpool Hospital and South West Sydney Local Health District / Neurovascular Imaging Lab, Clinical Sciences Stream, Ingham Institute, AUSTRALIA

## Abstract

**Background:**

Mechanical thrombectomy (MT) has become the gold standard care for treating acute ischemic stroke (AIS) due to large vessel occlusion. Emerging evidence suggests that understanding the composition of clots prior to intervention could be useful for the selection of neuroendovascular techniques, potentially improving the efficacy of treatments. However, current imaging modalities lack the ability to distinguish clot composition accurately and reliably. Since water content can influence signal intensity on CT and MRI scans, its assessment may provide indirect clues about clot composition. This study aimed to elucidate the correlation between water content and clot composition using human clots retrieved from stroke patients and experimentally generated ovine clots.

**Materials and methods:**

This study involved an analysis of ten clots retrieved from patients with AIS undergoing MT. Additionally, we created ten red blood cells (RBC)-rich and ten fibrin-rich ovine blood clots, which were placed in a human intracranial vascular model under realistic flow conditions. The water content and compositions of these clots were evaluated, and linear regression analyses were performed to determine the relationship between clot composition and water content.

**Results:**

The regression analysis in human stroke clots revealed a significant negative association between RBC concentration and water content. We also observed a positive correlation between water content and both fibrin and platelets in ovine blood clots.

Conclusion

We identified a significant inverse relationship between clot RBC concentration and water content. Accurate detection of this feature through diagnostic imaging could be beneficial for preoperative clot characterization and planning in MT for AIS.

## Introduction

Acute ischemic stroke (AIS) is a major cause of fatality and disability around the world [1]. A significant cause of cerebral infarction is large vessel occlusion (LVO), a condition in which major brain arteries are blocked, often leading to severe ischemic strokes. LVOs are associated with high rates of disability and mortality, making them a critical focus in stroke research and treatment [2,3]. Mechanical thrombectomy (MT), a procedure to remove the occluding clot, has become a gold standard treatment for ischemic stroke caused by LVO [4,5]. The most common MT methods involve the use of an aspiration catheter, stent retriever, or a combination of both [6]. While these techniques have demonstrated high rates of successful recanalization [7], achieving successful reperfusion with a single pass–deemed the “first-pass effect”–is associated with improved clinical outcomes and fewer procedure-related complications [8–10].

Clot pathologies play a crucial role in influencing the success of MT and achieving first-pass effect [11,12]. The stent retriever technique, for instance, demonstrates greater efficacy in removing red blood cells (RBC)-rich clots compared to fibrin-rich clots [13–15]. However, very soft RBC clots can be fragmented by the complete opening of the stent retriever [16]. On the other hand, fibrin-rich pathology can reduce the efficacy of stent retrievers, as fibrin-rich clots are not easily penetrated by the stent struts. In contrast, aspiration has demonstrated efficacy in removing very soft clots [16], although it may encounter difficulties with large, delicate clots or moderately stiff ones. A combined approach, harnessing- both an aspiration catheter and stent retriever- might offer a synergistic advantage, potentially elevating treatment efficacy. An in-vitro study showed that the combined technique achieved the highest success rates in removing stiff, calcified clots [17]. Consequently, determining clot pathology prior to MT could be useful for selecting the most effective initial treatment strategy [18–21].

Currently, there are no diagnostic imaging techniques that can reliably and consistently detail clot pathologies. Notably, the process of clot formation within blood vessels is complex and influenced by numerous factors, such as blood composition, the condition of the vessel wall, and blood flow [22,23]. Additionally, clots exhibit heterogeneous pathologies, with varying proportions of fibrin, RBCs, platelets, and white blood cells (WBC) as their primary compositions [24]. The hyperdense sign on CT or the blooming artifact observable on Susceptibility-Weighted Imaging (SWI) on MRI are useful for identifying RBC-rich clots [15,25,26]. However, there are no diagnostic imaging techniques that can elucidate clot characteristics reliably. Conversely, without predominant composition of RBCs, it is challenging to ascertain the extent of RBCs, fibrin, and platelets that make up the pathology from preoperative images. Recent studies have indicated that the complex behavior of animal clots correlates with their water content, and that differences in water content in animal in vitro clots might be detectable via CT [27,28]. Nonetheless, despite these findings, the relationship between water content and the pathology of human clots remains unknown.

In this study, we aimed to bridge this gap by examining the relationship between clot composition and water content in human clots harvested from patients with LVO, validating our findings in artificial animal blood clots.

## Methods

### Human clots from stroke patients

The study involving human blood clot collection was reviewed and approved by the Institutional Review Board (IRB) and the requirement for informed consent was waived for this study. We analyzed blood clots retrieved from AIS patients who underwent MT for LVO between July 2022 through February 2023. Indications for MT were determined according to the American Heart Association / American Stroke Association guidelines, and the procedure was performed after informed consent was obtained from each patient or the patient’s family [5]. The devices used were FDA-approved and commonly available devices, and the treatment strategy was determined by the neuroendovascular physicians [6].

The clots were divided into two sections: one portion was used to measure water content, while the other portion was utilized for pathology slides. For the pathological analysis, the slides were stained with Martius Scarlet Blue (MSB), and an image analysis software was employed to calculate the percentages of various clot components. The correlation between the clot constituents and their water content was then statistically analyzed.

### Ovine blood clot preparation

Ten RBC-rich clots and ten fibrin-rich clots were prepared using the modified Chandler loop method to create realistic clot analogs using ovine blood with sodium citrate from HemoStat Laboratories [29,30]. Polyvinyl chloride tubing (Length 31 cm and diameter 8 mm) was used for the loop and filled with the anticoagulated blood (9mL blood and 0.9mL calcium chloride). For RBC-rich clots, whole blood in the loops was then recalcified to reverse the anticoagulant effects by the addition of 10% calcium chloride. For fibrin-rich clots, plasma was prepared by centrifuging the whole blood carefully at low speed (550 g for 15 minutes at room temperature) and the fibrin-rich clot mixture was constituted by combining 99% of the plasma with 1% RBCs and the calcium chloride. The tubing, filled with either whole blood or plasma, was placed in the Chandler Loop apparatus and incubated at 37°C with a constant rotation speed of 10 rpm for 1 hour. Following incubation, the clots were carefully retrieved from the loops, ensuring minimal disruption to their structure. They were then stored in phosphate-buffered saline (PBS) at a temperature of 4°C until ready for use.

### In-vitro thrombectomy model

The in-vitro thrombectomy experimental system was constructed in accordance with the previously described protocol [31]. A pulsatile pump (Harvard Apparatus, MA, US) was connected to physiological flow loop and a silicone replica of human neurovascular anatomy, consisting of the internal carotid artery (ICA), posterior communicating artery, anterior communicating artery, and middle cerebral artery (MCA). PBS was circulated within the experimental system with the pulse rate at a 60 beats per minute (bpm), a flow rate of 240 mL/min for the ICA, a pressure of 120/80 mmHg, and a maintained temperature at 37°C. To induce occlusion in the MCA M1 segment, the clot was trimmed to 10 mm for RBC-rich clots and 7 mm for fibrin-rich clots. The clot analogs were then inserted into the cervical ICA and naturally positioned themselves within the M1 segment of the MCA under dynamic flow conditions. The clots were left to settle for 3 minutes to ensure stable and realistic occlusion. Following this settling period, the clot was extracted from the model for MSB staining by reversing the fluid flow in the system.

### Pathological analysis

The clots were fixed in a 4% paraformaldehyde solution for 24 hours and then longitudinally embedded in paraffin blocks. The clots were sliced into sections with a thickness of 4 μm. Selected sections, two per clot, were stained with a commercially available MSB kit (MSB Stain Kit RS4607-500, AVANTIK, US). High-resolution images of the MSB-stained clot slides were obtained using a digital slide scanner. These images were analyzed with Orbit Image Analysis software (www.orbit.bio), which utilizes machine learning algorithms to differentiate clot components, including RBC, WBC, fibrin, and platelet/others. The software performed segmentation of the clot images into these components and subsequent analysis calculating the percentage of each clot component.

### Water content measurement

The weight of the collected clots was measured with an analytical balance (METTLER TOLEDO, USA). Then, the clot was dried at 90°C for 8 hours to allow the water to evaporate sufficiently. The water content % was calculated using the formula: [(Initial wet weight−Dry weight)/Initial wet weight] ×100%, thus determining the proportion of water in the clot.

### Statistical analyses

For the analysis of the relationship between water content and clot composition, a linear regression analysis was conducted. The linearity of the relationship was assessed through scatter plots and the residuals were checked to ensure that assumptions of homoscedasticity and normality were met. The statistical model was as follows:

Water content = β0 + β1*(Composition of clot)

In the model, β0 represents the y-intercept (the estimated water content when the composition of the clot is zero), β1 is the regression coefficient (the change in water content for each unit change in the composition of the clot). The significance of the regression model was evaluated using an F-test, and the strength and direction of the relationship were determined by the coefficient of determination (R^2^) and the sign of the β1 coefficient, respectively. The regression coefficient (β1) was reported with a 95% confidence interval to provide an estimate of the uncertainty around the prediction. Student t-test was used for the comparison of water content between RBC-rich and fibrin-rich clots.

All data are presented as mean ± standard deviation. A p-value of <0.05 was considered statistically significant. All statistical analyses were performed using SPSS (v23 for Windows; SPSS Inc., Chicago, IL, USA) statistical software package.

## Results

### Human stroke clots

In this study, we analyzed human stroke clots from 10 patients with AIS who underwent MT for LVO, as shown in Table 1. The mean age of patients was 77 years, and 8 were male. Seven in ten patients had strokes due to cardioembolic etiology, while two in ten had strokes considered cancer-related (patient No.6 and No.10). One patient (No.5) had large artery atherosclerosis and was concurrently diagnosed with COVID-19. Intravenous thrombolysis with tissue plasminogen activator was administered to 7 patients. The average number of passes required for successful thrombectomy was 2.4.

**Table 1:** Breakdown of the patients underwent thrombectomy.

Patient No.	age	sex	past medical history	NIHSS	thrombolysis	occluded vessel	TOAST	thrombectomy technique	number of device pass	eTICI
1	46	Male	AF	16	1	Right MCA	Cardioembolic	combined	5	2b
2	74	Male	HT, HL, AF	18	0	Left MCA	Cardioembolic	stent retriever	1	3
3	68	Male	AF	10	1	Left MCA	Cardioembolic	combined	1	3
4	96	Male	CAD	18	1	Right MCA	Cardioembolic	aspiration	2	3
5	84	Male	COVID-19	18	1	Left ICA	Large artery atherosclerosis	aspiration	3	2c
6	78	Male	HT, HL, AF, CAD, CKD pancreatic cancer	22	0	Left ICA	Other (hypercoagulable state)	combined	6	2a
7	95	Female	HT, HL, AF	24	1	Left MCA	Cardioembolic	combined	1	2c
8	86	Male	HT, HL, CAD	23	1	Basilar artery	Cardioembolic	combined	1	3
9	58	Male	HT	5	1	Left ICA	Cardioembolic	combined	2	2b
10	84	Female	HT,HL,DM adenocarcinoma	4	0	Left MCA	Other (hypercoagulable state)	combined	2	3

Abbreviations: AF, Atrial Fibrillation; CAD, Coronary Artery Disease; CKD, Chronic Kidney Disease; COVID, Coronavirus Disease 2019; HL, Hyperlipidemia; HT, Hypertension; ICA, Internal Carotid Artery; MCA, Middle cerebral artery; MI, Myocardial Infarction; NIHSS, National Institutes of Health Stroke Scale; eTICI, extended Thrombolysis in Cerebral Infarction; TOAST, Trial of Org 10172 in. Acute Stroke Treatment.

**Fig 1:**
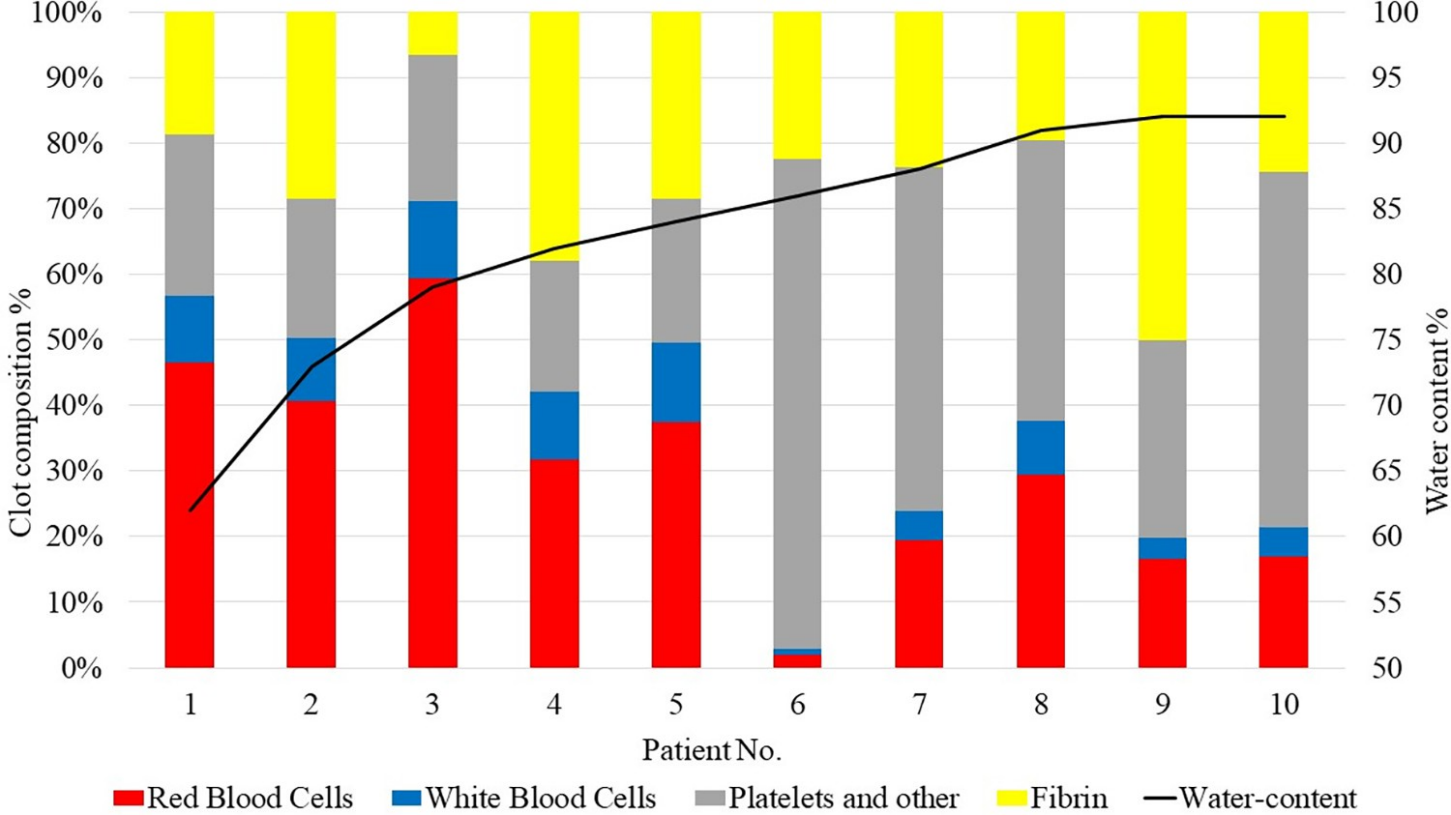
The clot composition and water content of human stroke clots. A bar graph illustrates the histological composition of human stroke clots, determined using Martius Scarlet Blue staining, with the following color representations: Red for red blood cell, blue for white blood cell, yellow for fibrin, and grey for platelet and others. The water content of each human stroke clot is concurrently shown in a line graph.

**Fig 1** demonstrates the heterogeneity in primary clot compositions among the patient cohort, arranged by ascending water content. On average, human stroke clots was consisted of 30.04% RBCs, 7.50% white blood cells, 36.43% platelets, and 26.04% fibrin. In patients with cancer-related strokes (patient No.6 and No.10), platelets were the predominant component of the clot material, constituting 54% and 75%, respectively. In contrast, cellular elements, including red and white blood cells, were the least, accounting for 21% and 3%, respectively. The results of the regression analysis are shown in **Table 2**, which reveals an inverse association between the proportion of RBCs and water content—a lower percentage of RBCs was significantly correlated with increased water content in the clots. Pearson correlation analysis further indicated a statistically significant relationship between the fibrin and platelets fraction in human clots and water content, with correlation coefficient of r = 0.643 (p = 0.045).

**Table 2:** Results of the regression analysis in human clots.

	β0	β1	P value	R^2^
Fibrin	76.049	26.309	0.364	0.104
Platelets and other	73.606	25.514	0.139	0.253
White Blood Cells	92.876	-133.084	0.098	0.305
Red Blood Cells	93.998	-36.944	0.040	0.427

### Ovine blood clots

**Fig 2:**
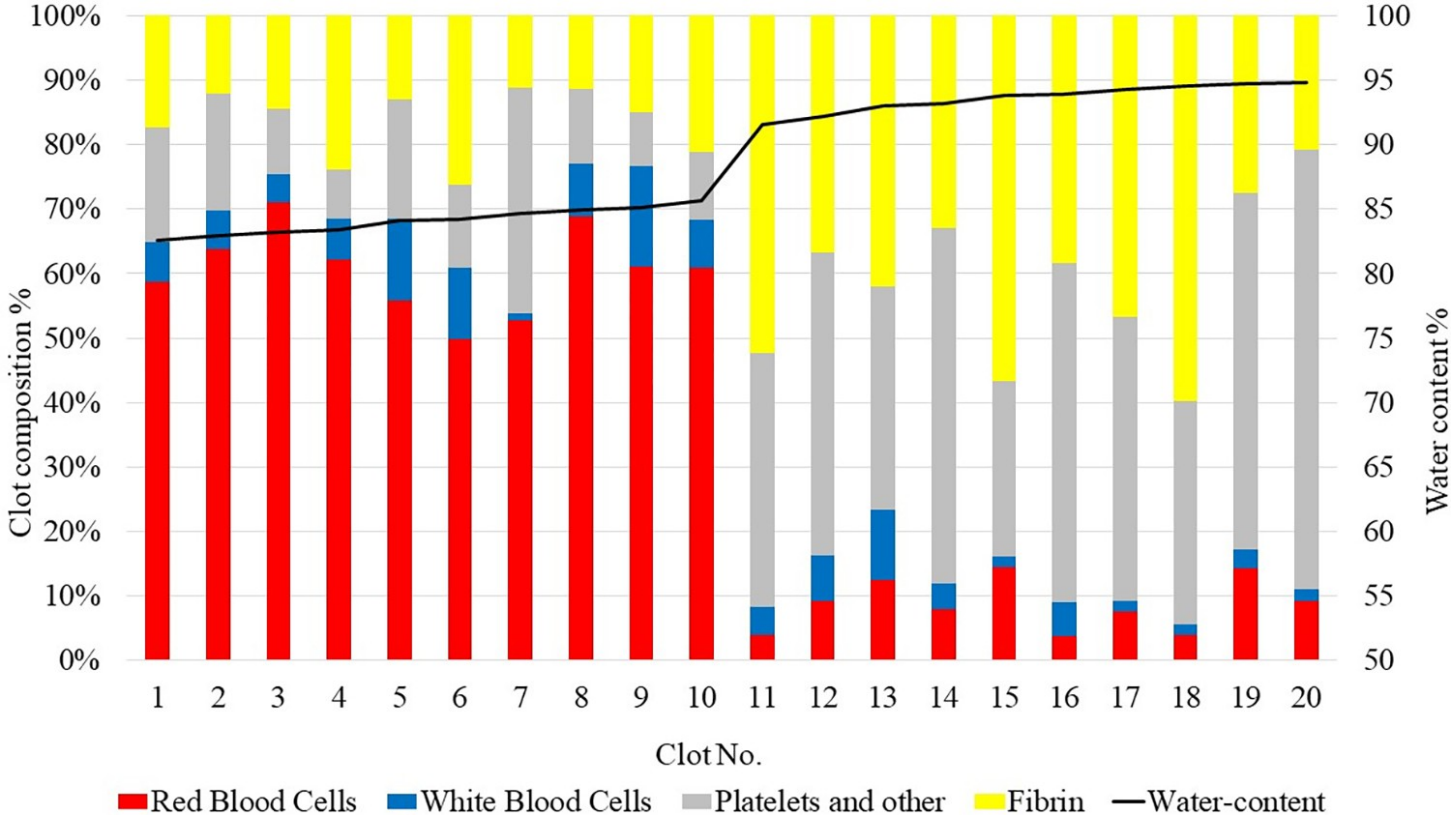
The clot composition and water content of ovine blood clots. A bar graph illustrates the histological composition of ovine blood clots, determined using Martius Scarlet Blue staining, with the following color representations: Red for red blood cell, blue for white blood cell, yellow for fibrin, and grey for platelet and others. The water content of each ovine blood clot is concurrently shown in a line graph.

**Fig 2** shows the composition and water content of ovine blood clots. Clots labeled 1 to 10 were formed as RBC-rich clots, whereas clots labeled 11 to 20 were created as fibrin-rich clots. The pathological analysis revealed that RBC-rich ovine clots presented with a notably higher proportion of RBCs at 60.50%, accompanied by 7.91% WBCs, 15.05% PLTs, and 16.55% fibrin on average. On the other hand, the fibrin-rich ovine clots demonstrated significantly lower RBC content at 8.68%, WBCs at 4.09%, a substantial amount of PLTs at 45.86% and fibrin at 41.37%. The average water content was significantly lower in the RBC-rich clots than in fibrin-rich clots, with values of 84.1±1.0% and 93.6±1.1%, respectively (P<0.001). **Table 3** summarizes the outcomes of the regression analysis, which demonstrated statistical significance of the regression model for all clot components, with the percentage of RBCs exhibiting the strongest predictive value. Similarly, Pearson correlation analysis revealed a statistically significant association between the fibrin and platelets fraction and water content, with a correlation coefficient r = 0.958 (p<0.001).

**Table 3:** Results of the regression analysis in animal clots.

	β0	β1	P value	R^2^
Fibrin	81.901	23.986	<0.001	0.585
Platelets and other	82.041	22.349	<0.001	0.711
White Blood Cells	92.409	-59.407	0.031	0.234
Red Blood Cells	94.943	-17.623	<0.001	0.921

## Discussion

In this study, we investigated the relationship between clot composition and water content in both human stroke clots and experimentally created ovine blood clots, demonstrating an inverse association between clot water content and RBC percentage. Furthermore, we observed a positive correlation between water content and both fibrin and platelet proportions in animal clots.

Our results are consistent with prior studies demonstrating an inverse relationship between water content and RBC composition in artificially created ovine blood clots [27]. Previously, it was shown that for every 1% increase in RBCs, there was a corresponding 0.26% reduction in water content. In the present study, an increase of 1% in RBCs resulted in a 0.37% decrease in human stroke clot water content and a 0.18% decrease in ovine blood clot water content. A potential reason for this inverse relationship between water content and RBC composition could be that RBC may contain a lower water content than other blood components, as detailed below.

Our study also identified a moderate positive correlation between water content and the presence of fibrin and platelets in line with the report by Gonzalez et al [27]. The fibrin network, comprising branched fibers, contributes to water retention due to its mesh-like protein structure [32,33]. However, while ovine blood clots showed a significant correlation between water content and fibrin component, this correlation was not significant in human stroke clots. Brown et al. demonstrated that stretching a clot modifies the arrangement of its mesh structure, leading to a decrease in water content and a reduction in volume [33]. Additionally, Ghezelbash et al. investigated the poroelastic property of a blood clot and identified that under deformation, water migrates in the porous medium of a blood clot [28]. These suggest that the water content in a blood clot is dynamic and can change as the clot deforms due to the movement of water within the clot’s structure. Given these insights, the limited correlations in our study may be due to the intrinsic properties and mechanics of these components within the clot.

Overall, our findings contribute to the understanding of the relationship between clot pathologies and their water content. A deeper insight into the water content of clots offers the potential to leverage this property in diagnostic imaging for the identification of specific clot compositions. The ability to predict clot features from preprocedural images could allow neuroendovascular physicians to select the optimal devices, increasing the chances of achieving first-pass success, and improving patient outcomes. There are several avenues of research ongoing to identify methods to detect clot characteristics preoperatively, including the use of machine-learning radiomics [34]. Based on our data, future research should prioritize utilizing readily available diagnostic imaging techniques, such as MRI, to potentially visualize the magnitude of water content within clots.

There are some limitations to this study. First, clots retrieved by MT are subjected to mechanical manipulation by the MT device, which can alter clot structure and its water content [28]. Secondly, the administration of thrombolysis in some patients may have influenced the pathological analysis due to its fibrinolytic effect. This study is a proof of concept and limited by the small number of cases, which restricted our ability to determine the impacts of imaging characteristics with CT or MRI and thrombolysis effects on clot composition. Such studies would significantly contribute to optimizing treatment strategies and improving patient outcomes in acute ischemic stroke management.

## Conclusion

This study identified RBC content as a primary determinant of clot water content. This observation holds promise for the innovation of advanced MRI and CT imaging aimed at preoperative clot characterization, which could aid in strategic planning in MT procedures for LVO in AIS.

## References

[1] Feigin VL, Brainin M, Norrving B, Martins S, Sacco RL, Hacke W, et al. World Stroke Organization (WSO): Global Stroke Fact Sheet 2022. Int J Stroke. 2022;17(1):18–29. doi: 10.1177/17474930211065917 . 34986727

[2] Mendelson SJ, Prabhakaran S. Diagnosis and Management of Transient Ischemic Attack and Acute Ischemic Stroke: A Review. JAMA. 2021;325(11):1088–98. doi: 10.1001/jama.2020.26867 . 33724327

[3] Goyal M, Menon BK, van Zwam WH, Dippel DW, Mitchell PJ, Demchuk AM, et al. Endovascular thrombectomy after large-vessel ischaemic stroke: a meta-analysis of individual patient data from five randomised trials. Lancet. 2016;387(10029):1723–31. Epub 20160218. doi: 10.1016/S0140-6736(16)00163-X . 26898852

[4] Smith EE, Saver JL, Cox M, Liang L, Matsouaka R, Xian Y, et al. Increase in Endovascular Therapy in Get With The Guidelines-Stroke After the Publication of Pivotal Trials. Circulation. 2017;136(24):2303–10. doi: 10.1161/CIRCULATIONAHA.117.031097 . 28982689

[5] Powers WJ, Rabinstein AA, Ackerson T, Adeoye OM, Bambakidis NC, Becker K, et al. Guidelines for the Early Management of Patients With Acute Ischemic Stroke: 2019 Update to the 2018 Guidelines for the Early Management of Acute Ischemic Stroke: A Guideline for Healthcare Professionals From the American Heart Association/American Stroke Association. Stroke; a journal of cerebral circulation. 2019;50(12):e344–e418. doi: 10.1161/STR.0000000000000211 . 31662037

[6] Yeo LLL, Jing M, Bhogal P, Tu T, Gopinathan A, Yang C, et al. Evidence-Based Updates to Thrombectomy: Targets, New Techniques, and Devices. Front Neurol. 2021;12:712527. Epub 20210909. doi: 10.3389/fneur.2021.712527 ; PubMed Central PMCID: PMC8459011. 34566856 PMC8459011

[7] Badhiwala JH, Nassiri F, Alhazzani W, Selim MH, Farrokhyar F, Spears J, et al. Endovascular Thrombectomy for Acute Ischemic Stroke: A Meta-analysis. JAMA. 2015;314(17):1832–43. doi: 10.1001/jama.2015.13767 . 26529161

[8] Zaidat OO, Castonguay AC, Linfante I, Gupta R, Martin CO, Holloway WE, et al. First Pass Effect: A New Measure for Stroke Thrombectomy Devices. Stroke; a journal of cerebral circulation. 2018;49(3):660–6. Epub 20180219. doi: 10.1161/STROKEAHA.117.020315 . 29459390

[9] Abbasi M, Liu Y, Fitzgerald S, Mereuta OM, Arturo Larco JL, Rizvi A, et al. Systematic review and meta-analysis of current rates of first pass effect by thrombectomy technique and associations with clinical outcomes. J Neurointerv Surg. 2021;13(3):212–6. Epub 20210113. doi: 10.1136/neurintsurg-2020-016869 ; PubMed Central PMCID: PMC9041815. 33441394 PMC9041815

[10] Jadhav AP, Desai SM, Zaidat OO, Nogueira RG, Jovin TG, Haussen DC, et al. First Pass Effect With Neurothrombectomy for Acute Ischemic Stroke: Analysis of the Systematic Evaluation of Patients Treated With Stroke Devices for Acute Ischemic Stroke Registry. Stroke; a journal of cerebral circulation. 2022;53(2):e30–e2. Epub 20211117. doi: 10.1161/STROKEAHA.121.035457 . 34784741

[11] Waqas M, Li W, Patel TR, Chin F, Tutino VM, Dossani RH, et al. Clot imaging characteristics predict first pass effect of aspiration-first approach to thrombectomy. Interv Neuroradiol. 2022;28(2):152–9. Epub 20210518. doi: 10.1177/15910199211019174 ; PubMed Central PMCID: PMC9131505. 34000868 PMC9131505

[12] Duffy S, McCarthy R, Farrell M, Thomas S, Brennan P, Power S, et al. Per-Pass Analysis of Thrombus Composition in Patients With Acute Ischemic Stroke Undergoing Mechanical Thrombectomy. Stroke; a journal of cerebral circulation. 2019;50(5):1156–63. doi: 10.1161/STROKEAHA.118.023419 . 31009342

[13] Yuki I, Kan I, Vinters HV, Kim RH, Golshan A, Vinuela FA, et al. The impact of thromboemboli histology on the performance of a mechanical thrombectomy device. AJNR American journal of neuroradiology. 2012;33(4):643–8. Epub 20111229. doi: 10.3174/ajnr.A2842 ; PubMed Central PMCID: PMC8050469. 22207297 PMC8050469

[14] Maekawa K, Shibata M, Nakajima H, Mizutani A, Kitano Y, Seguchi M, et al. Erythrocyte-Rich Thrombus Is Associated with Reduced Number of Maneuvers and Procedure Time in Patients with Acute Ischemic Stroke Undergoing Mechanical Thrombectomy. Cerebrovasc Dis Extra. 2018;8(1):39–49. Epub 20180115. doi: 10.1159/000486042 ; PubMed Central PMCID: PMC5836222. 29402828 PMC5836222

[15] Shin JW, Jeong HS, Kwon HJ, Song KS, Kim J. High red blood cell composition in clots is associated with successful recanalization during intra-arterial thrombectomy. PLoS One. 2018;13(5):e0197492. Epub 20180521. doi: 10.1371/journal.pone.0197492 ; PubMed Central PMCID: PMC5962078. 29782513 PMC5962078

[16] Kaneko N, Ghovvati M, Komuro Y, Guo L, Khatibi K, Ponce Mejia LL, et al. A new aspiration device equipped with a hydro-separator for acute ischemic stroke due to challenging soft and stiff clots. Interv Neuroradiol. 2022;28(1):43–9. Epub 20210505. doi: 10.1177/15910199211015060 ; PubMed Central PMCID: PMC8905075. 33951972 PMC8905075

[17] Johnson S, McCarthy R, Fahy B, Mereuta OM, Fitzgerald S, Gaudirc J, et al. Development of an in vitro model of calcified cerebral emboli in acute ischemic stroke for mechanical thrombectomy evaluation. J Neurointerv Surg. 2020;12(10):1002–7. Epub 20200103. doi: 10.1136/neurintsurg-2019-015595 . 31900353

[18] Garcia-Tornel A, Rubiera M, Requena M, Muchada M, Pagola J, Rodriguez-Luna D, et al. Sudden Recanalization: A Game-Changing Factor in Endovascular Treatment of Large Vessel Occlusion Strokes. Stroke; a journal of cerebral circulation. 2020;51(4):1313–6. Epub 20200214. doi: 10.1161/STROKEAHA.119.028787 . 32078495

[19] Choi MH, Park GH, Lee JS, Lee SE, Lee SJ, Kim JH, et al. Erythrocyte Fraction Within Retrieved Thrombi Contributes to Thrombolytic Response in Acute Ischemic Stroke. Stroke; a journal of cerebral circulation. 2018;49(3):652–9. Epub 20180126. doi: 10.1161/STROKEAHA.117.019138 . 29374103

[20] van Voorst H, Bruggeman AAE, Andriessen J, Hoving JW, Konduri PR, Yang W, et al. Prognostic Value of Thrombus Volume and Interaction With First-Line Endovascular Treatment Device Choice. Stroke; a journal of cerebral circulation. 2023;54(4):1056–65. Epub 20230313. doi: 10.1161/STROKEAHA.122.041606 . 36912141

[21] Boodt N, Snouckaert van Schauburg PRW, Hund HM, Fereidoonnezhad B, McGarry JP, Akyildiz AC, et al. Mechanical Characterization of Thrombi Retrieved With Endovascular Thrombectomy in Patients With Acute Ischemic Stroke. Stroke; a journal of cerebral circulation. 2021;52(8):2510–7. Epub 20210603. doi: 10.1161/STROKEAHA.120.033527 ; PubMed Central PMCID: PMC8312567. 34078112 PMC8312567

[22] Hathcock JJ. Flow effects on coagulation and thrombosis. Arterioscler Thromb Vasc Biol. 2006;26(8):1729–37. Epub 20060601. doi: 10.1161/01.ATV.0000229658.76797.30 . 16741150

[23] Lopez JA, Chen J. Pathophysiology of venous thrombosis. Thromb Res. 2009;123 Suppl 4:S30–4. doi: 10.1016/S0049-3848(09)70140-9 . 19303501

[24] Alkarithi G, Duval C, Shi Y, Macrae FL, Ariens RAS. Thrombus Structural Composition in Cardiovascular Disease. Arterioscler Thromb Vasc Biol. 2021;41(9):2370–83. Epub 20210715. doi: 10.1161/ATVBAHA.120.315754 ; PubMed Central PMCID: PMC8384252. 34261330 PMC8384252

[25] Froehler MT, Tateshima S, Duckwiler G, Jahan R, Gonzalez N, Vinuela F, et al. The hyperdense vessel sign on CT predicts successful recanalization with the Merci device in acute ischemic stroke. J Neurointerv Surg. 2013;5(4):289–93. Epub 20120522. doi: 10.1136/neurintsurg-2012-010313 ; PubMed Central PMCID: PMC4156587. 22619467 PMC4156587

[26] Rovira A, Orellana P, Alvarez-Sabin J, Arenillas JF, Aymerich X, Grive E, et al. Hyperacute ischemic stroke: middle cerebral artery susceptibility sign at echo-planar gradient-echo MR imaging. Radiology. 2004;232(2):466–73. Epub 20040623. doi: 10.1148/radiol.2322030273 . 15215546

[27] Velasco Gonzalez A, Buerke B, Gorlich D, Fobker M, Rusche T, Sauerland C, et al. Clot Analog Attenuation in Non-contrast CT Predicts Histology: an Experimental Study Using Machine Learning. Transl Stroke Res. 2020;11(5):940–9. Epub 20200114. doi: 10.1007/s12975-019-00766-z . 31933117

[28] Ghezelbash F, Liu S, Shirazi-Adl A, Li J. Blood clot behaves as a poro-visco-elastic material. J Mech Behav Biomed Mater. 2022;128:105101. Epub 20220129. doi: 10.1016/j.jmbbm.2022.105101 . 35124354

[29] Zeng Z, Nallan Chakravarthula T, Christodoulides A, Hall A, Alves NJ. Effect of Chandler loop shear and tubing size on thrombus architecture. J Mater Sci Mater Med. 2023;34(5):24. Epub 20230512. doi: 10.1007/s10856-023-06721-7 ; PubMed Central PMCID: PMC10182104. 37173603 PMC10182104

[30] Berndt M, Prothmann S, Maegerlein C, Oberdieck P, Zimmer C, Hegge B, et al. Artificial Stroke Clots: How Wide is the Gap to the Real World? World Neurosurg. 2018;110:e90–e9. Epub 20171026. doi: 10.1016/j.wneu.2017.10.090 . 29107162

[31] Kaneko N, Mashiko T, Ohnishi T, Ohta M, Namba K, Watanabe E, et al. Manufacture of patient-specific vascular replicas for endovascular simulation using fast, low-cost method. Sci Rep. 2016;6:39168. Epub 20161215. doi: 10.1038/srep39168 ; PubMed Central PMCID: PMC5156941. 27976687 PMC5156941

[32] Voter WA, Lucaveche C, Erickson HP. Concentration of protein in fibrin fibers and fibrinogen polymers determined by refractive index matching. Biopolymers. 1986;25(12):2375–84. doi: 10.1002/bip.360251214 . 3801589

[33] Brown AE, Litvinov RI, Discher DE, Purohit PK, Weisel JW. Multiscale mechanics of fibrin polymer: gel stretching with protein unfolding and loss of water. Science. 2009;325(5941):741–4. doi: 10.1126/science.1172484 ; PubMed Central PMCID: PMC2846107. 19661428 PMC2846107

[34] Hofmeister J, Bernava G, Rosi A, Vargas MI, Carrera E, Montet X, et al. Clot-Based Radiomics Predict a Mechanical Thrombectomy Strategy for Successful Recanalization in Acute Ischemic Stroke. Stroke; a journal of cerebral circulation. 2020;51(8):2488–94. Epub 20200720. doi: 10.1161/STROKEAHA.120.030334 ; PubMed Central PMCID: PMC7382538. 32684141 PMC7382538

