# Supplementary material for: Water content for clot composition prediction in acute ischemic stroke
Source: PLoS One. 2024 May 24;19(5):e0304520. doi: 10.1371/journal.pone.0304520 (PMC11125472; doi:10.1371/journal.pone.0304520)
Supplement: Attachment — Submitted filename: Response to reviewer.docx [file pone.0304520.s001.docx]

**Point-by-point response to the reviewers**

I'd like to extend my gratitude to both the editor and the reviewers for the time and effort spent reviewing our manuscript, as well as for the valuable feedback provided.

**Reviewer 1**

*the authors have performed a study on water contact in cerebral clots and found a correlation between water content and RBCs. the authors could maybe provide images pf the clot: CT images as well as microscopy in order to illustrate their findings.This will without doubt impact treatment based on imaging*

Thank you for your constructive feedback on our manuscript. As you mentioned, the imaging analyses would enhance the impact of our study by providing direct visual evidence of the correlation between CT images and clot pathology.

It is important to note that the present study is a proof of concept with a relatively small sample size of human clots and those pre-thrombectomy images were taken with not only CT but also MRI imaging. Therefore, due to the limited number of cases and the variability in imaging modalities, meaningful statistical analysis of imaging data was not feasible. We have added this in the limitation session in Discussion. We believe that future reports will include these relationship.

**Reviewer #2**

*The aim of this study was to gain insight into the possible quantitative relationship between water content and blood elements in clot composition. Human clots obtained from stroke patients and sheep clots obtained in the laboratory were analysed. Although the sample sizes were rather small, some significant associations were found. Notably, there was an inverse association between water content and red blood cell (RBC) content in human clots. Associations between clot composition and medical image features were not evaluated, limiting the translational impact of the results. In addition, the following points should be addressed.*

*1.- Abstract. Lines 41-43 contain two redundant sentences. They should be reformulated into a single sentence stating the inverse association between water and RBC content. Line 49 should state the nature of the link between water and RBCs.*

Thank you for your insightful feedback. Regarding the observation of redundant sentences in lines 41-43, we have carefully reviewed the parts and now revised these lines.

*2.- Ovine Blood Clot Preparation. Line 114. Which anticoagulant was used?*

We acknowledge the importance of this detail for the reproducibility and clarity of our methods. Sodium citrate was the anticoagulant and has been amended in Line 111.

*3.- Table 1 should show the device(s)s used in each case (i.e., stent retriever, aspiration or combined).*

We appreciate your suggestion to include the types of devices used in each case in Table 1. we have revised the Table 1 accordingly.

*4.- Although the number of human clots is small, please try to assess a possible association between water/RBC content and the number of device passes (or first effect occurrence) as a measure of clot resistance to MT. If possible, try to include the effect of previous thrombolysis and/or the MT method (device(s)) in the statistical model.*

Thank you for your suggestion. We recognize the potential value of such an analysis of clot resistance to MT. However, due to the limited number of human clots available in our proof of concept study, it was not feasible to do this analysis with sufficient statistical power. We have added this in the limitation session in Discussion. We believe that future reports will include these relationship.

*5.- Table 2. Since fibrin and platelets are functionally related in clot formation, some clot composition studies use fibrin+platelets as a clot fraction. Please consider assessing the possible association between fibrin+platelets fraction and water content.*

In response to your suggestion, we have revised our manuscript to include an analysis of the fibrin+platelets fraction in relation to water content. This addition will strengthen the manuscript by offering a broader perspective on how clot composition is related with water content.

*6.- Ovine Blood Clots. Lines 206-207. Should read “The average water content was significantly lower in RBC-rich clots than in fibrin-rich clots”.*

We have reviewed the mentioned lines and accordingly, we have revised lines 206.

*7.- Study limitations. Lines 252-253. See comment in point 4 regarding the inclusion of the effect of prior thrombolysis in the statistical model.*

This study is a proof of concept and limited by the small number of cases, which restricted our ability to determine the impacts of imaging characteristics with CT or MRI and thrombolysis effects on clot composition. Such studies would significantly contribute to optimizing treatment strategies and improving patient outcomes in acute ischemic stroke management. We have added this in the limitation session in Discussion.
