# Supplementary material for: Water content for clot composition prediction in acute ischemic stroke
Source: PLoS One. 2024 May 24;19(5):e0304520. doi: 10.1371/journal.pone.0304520 (PMC11125472; doi:10.1371/journal.pone.0304520)
Supplement: Attachment — Submitted filename: Response to reviewer2.docx [file pone.0304520.s002.docx]

**Point-by-point response to the reviewers**

I'd like to extend my gratitude to both the editor and the reviewers for the time and effort spent reviewing our manuscript, as well as for the valuable feedback provided.

**Reviewer #2**

*Abstract. It should be clearly stated that there was a negative/inverse association between RBC and water contents, both in results (lines 41-42) and conclusion (line 46).*

Thank you for your constructive feedback on our manuscript. We have revised the abstract to explicitly state the negative/inverse association between RBC and water contents in both the results and conclusion sections as suggested.

**Reviewer #3**

*Thank you for the opportunity to review this manuscript. The authors addressed most of the comments of the reviewers and revised the manuscript accordingly.*

*However, I believe that Line 49 in the Abstract was not changed to reflect the nature of the link between water and RBCs as requested by Reviewer #2 (Point 1).*

We appreciate your suggestions. I have edited as described above.

*Also, I would like to ask the authors to add a short paragraph that summarizes the average for each clot component including human clots and ovine clot analogs.*

We have added the average composition data for each clot component, including human clots and ovine clot analogs, into each part in the results section. This addition provides a comprehensive overview of the clot compositions, thereby strengthening the findings presented in our study.
